# User-Centered Design of a Dementia-Friendly Privacy Policy for the FindMyApps Intervention

**DOI:** 10.1093/geroni/igab046.2256

**Published:** 2021-12-17

**Authors:** David Neal, Yvonne Kerkhof, Teake Ettema, Karin Dijkstra, Rose-Marie Dröes

**Affiliations:** 1 Amsterdam UMC, Amsterdam, Noord-Holland, Netherlands; 2 Saxion University of Applied Sciences, Deventer, Overijssel, Netherlands

## Abstract

The ability of people with dementia and their caregivers to successfully navigate online environments is increasingly important to their social health. However, uncertainty about privacy online is an important barrier. Theoretically, access to published privacy policies should allow users of websites or software applications to make informed decisions. In practice, such documents are often complicated texts, and consequently even less accessible to people with cognitive impairment than to the general population. We present results from a multi-stakeholder, user-centred design process, towards an accessible alternative: a ‘dementia-friendly privacy policy’. Three design sprints took place in 2021, led by participants of the ‘Smart Solutions Semester’ at Saxion University of Applied Sciences in the Netherlands, in collaboration with cognitively unimpaired laypeople, people with dementia, informal caregivers, and expert stakeholders. Outputs were specifications for the solution, low-fidelity prototypes and high-fidelity prototypes, respectively. The dementia-friendly privacy policy is now ready for implementation and further evaluation.

